# Draft genome sequence of *Pseudomonas aeruginosa* DG-1: a strain isolated from heavily petroleum-contaminated soil with petroleum degradation ability

**DOI:** 10.1128/mra.00021-25

**Published:** 2025-09-04

**Authors:** Zhineng Wu, Yaduo Yang, Linhao Kang, Quanli Man, Hanjie Zu, Xiaodong Ma

**Affiliations:** 1 School of Energy and Environmental Engineering, Hebei University of Technology 726991 , Tianjin, China; The University of Arizona, Tucson, Arizona, USA

**Keywords:** *Pseudomonas aeruginosa*, petroleum, degradation

## Abstract

We present the draft genome sequence of a petroleum-degrading bacterium *Pseudomonas aeruginosa* DG-1. The genome size of strain DG-1 is 6,734,315 bp with a GC content of 66.07%, which contains 6,213 genes, including crude oil degradation, biosurfactant synthesis, and quorum sensing, providing valuable insights into the mechanisms of petroleum biodegradation.

## ANNOUNCEMENT

Petroleum pollution necessitates sustainable remediation strategies, with microbial biodegradation emerging as an eco-friendly solution (1–3). *Pseudomonas aeruginosa* DG-1, a strain isolated from DaGang Oilfield soils in China (117°43′34″ E, 38°77′34″ N), demonstrates exceptional petroleum degradation capabilities. Petroleum-contaminated soil (10 g) was inoculated into 90 mL of mineral salt medium (MSM) containing 1% (vol/vol) crude oil and incubated at 30°C with 150 rpm for 7 days. Following sequential transfers to fresh MSM containing progressively increased crude oil concentrations (1% increments per transfer), the culture was enriched through five successive passages up to 5% (vol/vol) crude oil. A single bacterial colony (designated DG-1) was isolated from the final enrichment using solid lysogeny broth (LB) plates. The gram-negative strain formed distinctive yellow translucent colonies with irregular margins and produced water-soluble green pigments. Taxonomic characterization identified DG-1 as a *P. aeruginosa* strain, subsequently named *P. aeruginosa* DG-1.

A single DG-1 colony was inoculated into 100 mL LB medium (30°C, 150 rpm, 24 h) to logarithmic growth phase (OD_600_ = 1.0), and the genomic DNA was extracted using a magnetic bead-based bacterial DNA extraction kit (Majorbio, China). The same DNA was sequenced via PacBio Sequel IIe and NovaSeq X Plus platforms (Majorbio Biomedical, China). For Illumina, DNA was fragmented to ~400 bp (Covaris M220), which was subsequently used to construct libraries with the NEXTFLEX Rapid DNA-Seq Kit. The platform generated 1.39 Gb of raw bases and 1.26 Gb of clean data, with 99.3% genome coverage after sequencing. For PacBio, genomic DNA was fragmented at ~10 kb, which were circularized into SMRTbell templates (SMRTbell prep kit 3.0), and 43,589 reads were generated after sequencing (N50 = 9,444 bp, genome coverage = 99.85%). The manufacturer’s recommendations were used for all kits used. Raw data were quality-controlled with Fastp v0.23.0. The Illumina and PacBio HiFi reads were assembled using Unicycler v0.4.8 (4), which produced a 6,734,315 bp circular genome (GC: 66.07%) and polished with Pilon v1.22. Gene prediction identified 6,213 CDS (Glimmer/Prodigal [5]), 63 tRNAs (tRNAscan-SE v2.0 [6]), and 12 rRNAs (Barrap v0.9). Functional annotation utilized the NCBI Prokaryotic Genome Annotation Pipeline (PGAP v6.9) (7), supplemented by the Kyoto Encyclopedia of Genes and Genomes (KEGG), the Cluster of Orthologous Groups of proteins (COG), Gene Ontology (GO), Non-Redundant Protein Database (NR), and Protein Family Analysis and Modeling (Pfam).

Notably, strain DG-1 possesses genes involved in crude oil degradation (such as *alk*M, *pca*G) (8), biosurfactant and biofilm synthesis (such as *rhl* gene cluster) (9), and quorum sensing (*las*R-*las*I and *rhl*R-*rhl*I) (10), thereby providing significant insights into its biodegradation mechanisms to petroleum.

## Data Availability

The draft genome sequence of Pseudomonas aeruginosa DG-1 has been deposited in GenBank under the accession number CP176811, BioProject number PRJNA1201839, and BioSample number SAMN45944935. The raw sequence reads are available under Sequence Read Archive accession numbers SRR32108754 and SRR32076116 (PacBio).
